# Outcome of a newborn hearing screening program in a tertiary hospital in Malaysia: the first five years

**DOI:** 10.4103/0256-4947.75774

**Published:** 2011

**Authors:** Amirozi Ahmad, Irfan Mohamad, Suzana Mansor, Mohd Khairi Daud, Dinsuhaimi Sidek

**Affiliations:** From the Department of Otorhinolaryngology-Head and Neck Surgery, School of Medical Sciences, Universiti Sains Malaysia Kota Bharu, Kelantan, Malaysia

## Abstract

**BACKGROUND AND OBJECTIVE:**

Universal newborn hearing screening (UNHS) was started in the Hospital Universiti Sains Malaysia (HUSM) in January 2003. To comply with international standards, we determined the outcome of the newborn hearing screening program for the first 5 years of its implementation, from January 2003 to December 2007.

**METHODS:**

The program screened all infants who were delivered in HUSM. In a retrospective review, the outcomes in terms of coverage, prevalence of hearing impairment, referral rate for each screening, age at detection of hearing impairment and at hearing aid-fitting were analyzed.

**RESULTS:**

Ninety-eight percent of newborns were screened. The study included 16 100 randomly selected newborns. The initial screening referral rate was 25.5%. The prevalence of default for second and third screening was 33.9% and 40.7%, respectively. The mean (SD) age at detection of hearing impairment was 3.3 months (0.86). The mean (SD) age at fitting of a hearing aid was 13.6 (4.8) months. The prevalence of hearing impairment was 0.09%.

**CONCLUSION:**

A newborn hearing screening program is an important tool for early diagnosis and treatment. Even though the prevalence of hearing impairment may be low, the problem needs to be addressed early as the development of infants requires normal hearing.

Prevalence figures for permanent congenital sensorineural hearing loss are 1.5 to 2.2 per 1000 live births,^1^ making it the most frequently occurring birth defect. However, hearing loss is not readily detectable by routine clinical procedures or behavioral observation, although parents often report a suspicion of hearing loss, inattention or unresponsiveness to sound before hearing loss is confirmed.^2^,^3^

The mean age that hearing impairment is detected has decreased from 12 to 24 months to 3 to 6 months since introduction of newborn hearing screening programs in the United States.^4^ Moreover, the mean age at which infants receive hearing aids has been reduced from 13 to 16 months before the programs began to 5 to 7 months.^5^ The Joint Committee on Infant Hearing 1994 position statement endorsed the goal of universal detection of infants with hearing loss and encouraged continuing research and development to improve methodologies for identification of, and intervention for, hearing loss.^6^

In Malaysia, audiological and intervention services for the hearing-impaired children have been slowly developing since the early 1990s. A few hospitals have been implementing hospital-based newborn hearing screening since the early 2000s. The implementation of universal newborn hearing screening in Malaysia has been supported by the awareness of the negative impacts of late detection of permanent congenital hearing loss and the positive impact of early intervention on language, cognitive, educational and social development skills of the growing infant.^7^

## METHODS

The objective of this retrospective study was to assess the outcome of the newborn hearing screening program at Hospital Universiti Sains Malaysia (HUSM) within the first 5 years of its implementation, from 1 January 2003 to 31 December 2007. We sought to evaluate the coverage, prevalence, the initial screening referral rate, the number of defaulters, the age at detection of hearing impairment and age at hearing aid-fitting. A sample of all newborns delivered within the study period was identified by systematic random sampling that involved taking every other patient (alternating) from the hearing screening records of the Hospital Universiti Sains Malaysia (HUSM).

The registry records contained data for the first, second and third hearing screen. The medical records of newborns who failed the third screening were traced and all data analyzed. In our center, the first screening was done in the ward by trained personnel (technician, staff nurse, ward attendants) supervised by an audiologist. Both ears were cleaned prior to every screening. The first screening was done using distortion product otoacoustic emissions (DPOAE) and Bio-Logic Audx none with a frequency of 2 kHz, 3 kHz and 4 kHz. The screening was done at the bedside where the newborns usually slept beside their mothers. If the surroundings were too noisy, the screening was done in a quiet room. Screening was usually done after feeding. The second screening was also done using the otoacoustic emissions with the same frequency. This screening was done in a sound-treated room in the otorhynolarynology clinic by technicians or audiologists. The third screening was done and the diagnosis of hearing impairment confirmed by an audiologist by using a diagnostic auditory brainstem response. Defaulters are babies that did not re-appear for the second or third screening.

## RESULTS

Of the 33 427 delivered babies in HUSM, 32 745 (98%) were screened and 16 100 newborns were selected for study by systematic random sampling, including 8255 (51%) boys and 7845 (49%) girls; 97.5% were Malays, reflecting the population of the state. The mean (SD) age at detection of hearing impairment was 3.3 months (0.86). The mean (SD) age at fitting of a hearing aid was 13.6 (4.8) months. The prevalence of hearing impairment was 0.09%.

**Figure 1:**
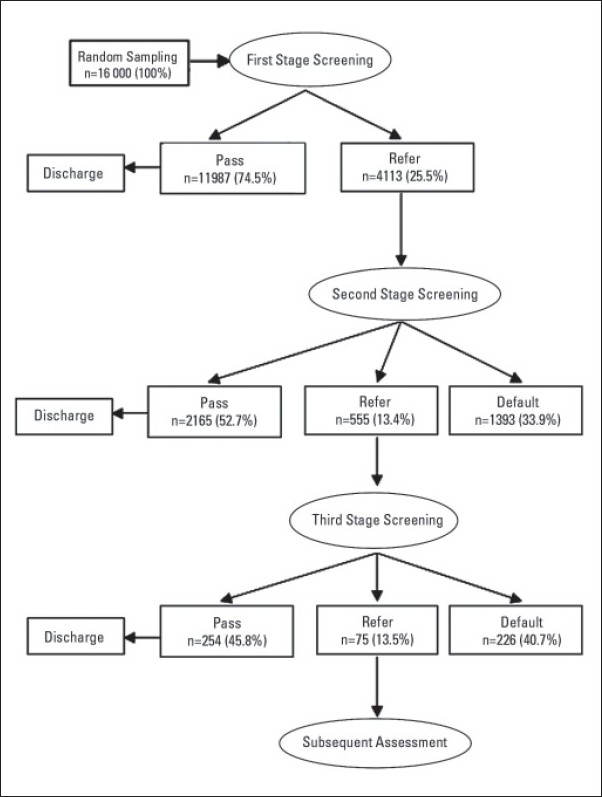
Flowchart for the evaluation of the newborn hearing screening program.

The percentage of newborns who passed, were referred or failed is shown in **Figure 1**. That percentage varied by year (**Figure 2a, b, c**).

**Figure 2a:**
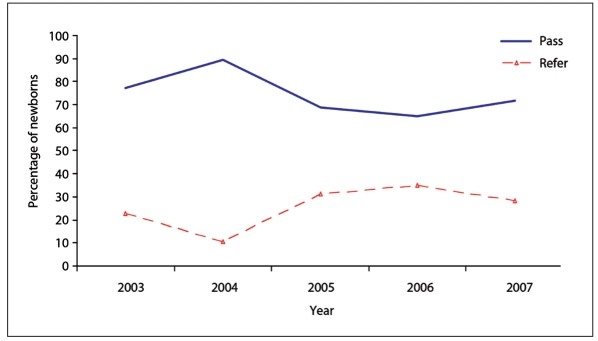
Results by year for first screening

**Figure 2b:**
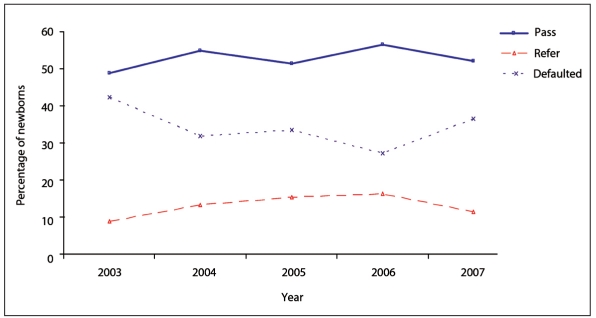
Results by year for second screening.

**Figure 2c:**
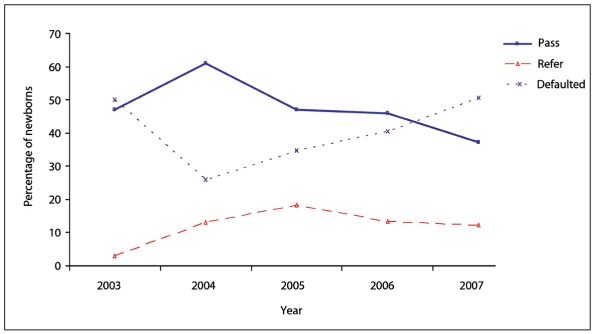
Results by year for third screening.

## DISCUSSION

Universal newborn hearing screening (UNHS) has been widely accepted and is being implemented worldwide. UNHS is designed to identify, as early as possible, congenital and acquired hearing loss. Early identification of hearing loss is imperative to prevent problems related to speech, language, social life and schooling from occurring at a later stage in life. This program requires all babies to be screened before discharge from the hospital. However, despite medical advances in screening instruments, UNHS is still restricted to developed countries. This is mostly due to the cost of the instruments and the manpower used in the screening program.

The coverage of newborns screened in our study was 98%. This percentage is high and achieves the quality indicator of screening established by the Joint Committee on Infant Hearing (JCIH), which is “more than 95%.”^8^ This percentage is also higher than that found in the two studies in Malaysia, which reported 89.2% and 84.6% coverage in the first screening.^9^,^10^ Good coverage for newborn hearing screening is very important in order not to miss any babies that might have hearing loss. It should cover all babies in the postnatal wards and intensive care. At our center, results from all wards and intensive care were recorded in a “first screening” book to avoid any misunderstandings, mistakes and missing data during the process of storing the data. The data was regularly checked by the screening staff to avoid any overlapping or duplication. Newborns that were delivered outside HUSM, but referred to intensive care were noted. Records are easily found and checked using this system. Newborn hearing screening was done everyday, including weekends.

The prevalence of hearing impairment detected during this study was only 0.09%, which is lower than that of other studies because of the high default rates among the newborns after they failed the third screening. The default rates were also high for subsequent follow-ups, which made confirmation of the hearing impairment difficult. The number of newborns who continued the follow-up until the diagnosis was made was small. These problems need to be addressed and solutions sought. The rate of defaults affects the accuracy of the true figure for prevalence.

In Malaysia, Abdullah et al found that the prevalence of hearing loss was 0.42% (16/3762).^9^ In our previous study, hearing screening in high-risk neonates revealed a total of 1% with hearing loss.^11^ From the published reports, the prevalence of mild-to-profound hearing loss is between 1.1 and 6.0 per 1000 live births.^12^–^14^ Another study by Narrigan in 2000 showed that an admission to intensive care for more than two days increased the risk of getting the hearing impairment to 10-fold.^15^

The initial screening referral rate in our study was 25.5%, which is higher compared to that found in related studies done in Malaysia.^9^,^10^ Many factors could have contributed to this result. Studies have shown a relatively high failure rate when screening is done at an early age. This has been attributed to obstruction of the ear canal with vernix, debris and amniotic fluid, which gradually disappear over the first few days of life.^16^ To get a lower referral rate in the first screening, it is best to screen after 24 hours. This has been supported by a study done under the Rhode Island Hearing Assessment Project (RIHAP) in neonates screened before 24 hours of age. They found that if they screened the babies after 24 hours of age, the pass rate was increased from 70% to 82%.^17^ Gabbard et al found that younger newborns had a higher referral rate than the older newborns when screened using otoacoustic emissions (OAE).^18^ The high failure rate in the initial screening might have been due to the early screening.

In another study, OAE was reported to have a high false-positive rate (about 15%) at the initial screening on the first day, which was then reduced by about 50% with each repeated screening.^19^ In our study, it is difficult to ascertain the false-positive rate because the default rate among initial ‘positive’ babies was very high. The false-negative rate was also difficult to ascertain because all the “negative” babies were discharged from follow-up in our center. These factors (high default and early discharge) may have contributed to the limitations of this retrospective study.

Another factor that can cause a high failure rate is the site of the screening test. Brass et al advocated that the screening be done in a quiet or soundproof room.^20^ In our program, the screening was mostly done in the ward with the baby beside the mother on the bed. OAE was used as the initial screening tool because it is technically easier and faster to perform and can accommodate a large number of patients. Besides, OAE is cheaper than auditory brainstem response, and this makes OAE more cost-effective as a screening tool. For greater accuracy, we use ABR for patients undergoing a third screening, as the sample for the third screening is much smaller and because OAE might miss patients with auditory neuropathy.^21^

The default rate in the second and third screening in our study was 33.9% and 40.7%, respectively. The reasons for the occurrence of these problems should be thoroughly investigated in order to improve the newborn hearing screening program in HUSM. Other similar studies in Malaysia also showed a high percentage of defaulters for both second and third screenings.^9^,^10^ This would reduce the effectiveness of the program because it reduces the detection of newborns with hearing loss or newborns that are hard of hearing. The high default rate fails the quality indicator established by JCIH. These high default rates or poor follow-up rates are attributed to numerous factors. Mukari et al sent questionnaires to 314 parents who failed to bring their babies for follow-up after failing the initial screening. Of 314 parents, 158 (50.32%) parents responded. They found that four factors contributed to poor follow-up rates: Lack of communication between the parents and screening personnel, weakness of the protocol for fixing follow-up appointments, lack of parents’ awareness regarding hearing loss and the need for early intervention, and problems in transportation.^10^

Despite that, another aspect that needs to be considered is parental concern. This is very important because false-positive results are inevitable. A false-positive result can cause unnecessary worries to the parents. Vohr et al found that mothers whose infants did not pass the routine newborn hearing screening described significantly greater worry about hearing screening than mothers whose infants had been screened, but who had not yet been informed of their infant’s screening result.^16^ More studies need to be done to assess the component of parental concern.

The objective of UNHS is to identify babies who have hearing loss and to provide necessary intervention as soon as possible. In this study, the age when hearing impairment was detected using diagnostic ABR ranged from 2.4 to 5.2 months (mean, 3.3 months; SD, 0.86). The age at hearing aid–fitting in this study ranged from 5 months to 18 months (mean, 13.6; SD, 4.8). One of the newborns detected at 5 months had successfully undergone cochlear implant. The success of a newborn hearing screening program depends on many components, such as commitment from various disciplines, regular evaluation of the program and efforts towards creating public awareness about hearing loss and early intervention. Many published studies on universal newborn hearing screening have demonstrated that this program is feasible and beneficial. At HUSM, the newborn hearing screening program, still needs regular evaluation to ensure quality improvement.
